# Novel data on growth phenotype and causative genotypes in 29 patients with Morquio (Morquio-Brailsford) syndrome from Central-Eastern Europe

**DOI:** 10.1007/s13353-019-00491-1

**Published:** 2019-03-30

**Authors:** Aleksandra Jezela-Stanek, Agnieszka Różdżyńska-Świątkowska, Anna Kulpanovich, Elżbieta Ciara, Jolanta Marucha, Anna Tylki-Szymańska

**Affiliations:** 1 Department of Genetics and Clinical Immunology, National Institute of Tuberculosis and Lung Diseases, Warsaw, Poland; 2 Anthropology Laboratory, The Children’s Memorial Health Institute, Warsaw, Poland; 3 National Medical Center “Mother and Child”, Ministry of Health, Orlovskaya Str, 66, Korpus 8, Offis 11, 220053 Minsk, Belarus; 4 Department of Medical Genetics, The Children’s Memorial Health Institute, Warsaw, Poland; 5 Department of Rehabilitation, The Children’s Memorial Health Institute, Warsaw, Poland; 6 Department of Paediatric, Nutrition and Metabolic Diseases, The Children’s Memorial Health Institute, Al. Dzieci Polskich 20, 04-730 Warsaw, Poland

**Keywords:** Morquio syndrome A, Mucopolysaccharidosis type IVA, Anthropometric features, Genotype-fenotype analysis

## Abstract

Mucopolysaccharidosis type IVA, also known as Morquio (Morquio-Brailsford) syndrome results from accumulation of keratan sulfate (KS) and chondroitin-6-sulfate (C6S), whereas the primary cause is mutations in the gene encoding galactosamine (*N*-acetyl)-6-sulfatase (GALNS). Phenotypically it seems to be a well-defined condition, with two main clinical forms: mild (attenuated) and severe, which are determined based on a combination of symptoms, i.e., enzymatic activity of GALNS, age of onset, and symptom severity. Nevertheless, the natural history of MPSIVA in relation to specific anthropometric parameters (growth, head circumference, body proportions, and face phenotype) is not precisely characterized. The aim of our work was to analyze the aforementioned anthropometric parameters, including correlation to molecular data (causative GALNS mutations).

## Introduction

Mucopolysaccharidosis type IVA (MIM #253000) is also known as Morquio syndrome or Morquio-Brailsford syndrome, after Uruguayan physician Luis Morquio, and British radiologist James Frederick Brailsford who described the condition in 1929 (Morquio 1929; Brailsford 1929). The observed clinical features result from accumulation of keratan sulfate (KS) and chondroitin-6-sulfate (C6S), whereas the primary cause is mutations in the gene encoding galactosamine (*N*-acetyl)-6-sulfatase (*GALNS*), located at 16q24.3 (Baker et al. 1993). According to Human Gene Mutation Database Professional (HGMD), 334 *GALNS* variants are currently mentioned (access 2018-10-17), including 332 disease-causing mutations and two of unknown clinical significance. The incidence of MPS IVA is approximately 0.22 per 100,000 births (range 0.07 to 1.32) (Poorthuis et al. 1999; Tomatsu et al. 2013).

The above-listed glycosaminoglycans (GAGs) cumulate in a variety of tissues but skeletal and ligament involvement are those which correspond to the most recognizable characteristic of Morquio syndrome (Neufeld and Muenzer 2001). At approximately 1–3 years of age, short stature may be noted, primarily due to a shortened neck and trunk, and is accompanied by significant joint hypermobility and ligamentous laxity (Tomatsu et al. 2011). Pectus carinatum (sternal protrusion) and genu valgum (knock-knee deformity) are other most common features. The clinical severity varies however among patients and MPS IVA manifests as severe or attenuated forms, depending on the amount of residual enzyme activity. In the severe forms, linear growth after 6–7 years of age is minimal, and a high mortality result from respiratory complications (as airway obstruction, sleep-disordered breathing, and restrictive lung disease) is observed (Hendriksz et al. 2015). A number of affected persons present also with atlantoaxial instability leading to cervical myelopathy, spinal canal stenosis, and spinal cord compression.

Contrary, mildly ill patients usually survive into the seventh decade, may suffer however from disability as a consequence of musculoskeletal involvement which may be associated with pain and arthritis in some. Mild corneal opacities, enamel hypoplasia, progressive hearing loss, and valvular heart disease may also occur in Morquio syndrome.

The aim of our study was to delineate the anthropometric phenotype in Morquio syndrome (MPS IVA), and also to correlate it with underlying genotype; such studies cannot be found in the literature.

## Material and methods

### Study group

Our group includes 29 patients from 21 families (8 siblings; 13 females and 16 males; age at diagnosis ranges from 1 to 28 years). Four affected from one family (individuals 1, 2, 14, and 15) were reported earlier (Bunge et al. 1997; Tylki-Szymanska et al. 1998). There are also two other affected males mentioned in this paper, they are however not included herein because of limited clinical data. Except four patients from two families (3, 4 and 10, 11) from Belarus (National Medical Center “Mother and Child Minsk), all others were from Poland (The Children Memorial Health Institute, Warsaw).

The diagnosis of mucopolysaccharidosis type IVA was based on clinical features, confirmed in biochemical (*N*-acetylgalactosamine-6-sulfatase, GALNS, activity) and molecular tests (pathogenic variants in *GALNS* gene). From the diagnosis onward, the pediatric patients underwent anthropometric assessment with growth evaluation, including length/height, chest and head circumference, as well as craniometric analyses.

### Anthropometric (growth and head circumference) assessment

The mean birth body height and weight were calculated. One patient was measured a few times (ranged from two to 13 times) while monitoring was performed. The number of measurements for one parameter ranged from 13 to 15, and time between measurements ranged from 6 months to 9 years.

Anthropometric measurements included: body height and weight; length of the head and neck; trunk length; lower and upper extremity lengths; shoulders, chest, and hip width; chest depth; chest circumference; head circumference; head length and breadth; and occipital, forehead, bizygomatic, and bigonial breadths. All measurements were taken at the Anthropology Laboratory, The Children’s Memorial Health Institute (CMHI), according to the standard anthropometric techniques (Martin and Saller 1957). Until the age of 3 years, length was measured in the supine position using the liberometer (accuracy to 1 mm). The same measurements of the older children were performed as standing height, using the stadiometer (accuracy to 1 mm). Weight was measured using electronic scale accurate within 0.05 kg. A non-stretchable tape was used to asses head and chest circumference (accuracy to 5 mm). The same anthropologist performed all assessments. All measurements were standardized for age and gender using Polish reference charts (Palczewska and Niedźwiecka 2001).

A statistical analysis was performed using Statistica, v.8 (StatSoft). Shapiro-Wilk and Kołmogorov-Smirnov tests were used to assess of sample normality. Two-tailed *t* test was used to compare the mean values for birth body length and weight between children with MPS type IVA and the healthy population. The significance level was assumed at 0.05.

### Molecular study

Molecular analysis was performed after obtaining informed consent from the patients’ parents or legal guardians. The direct DNA sequencing was applied to identify molecular defects in GALNS gene. The analysis was performed at the MedGen Medical Center, according to established laboratory protocols.

The nomenclature of molecular variants follows the Human Genome Variation Society guidelines (HGVS, www.hgvs.org/mutnomen) according to GALNS reference sequence NM_000512.4 and NP_000503.1, followed the Human Gene Mutation Database (HGMD Professional, https://portal.biobase-international.com/hgmd/) and ClinVar (https://www.ncbi.nlm.nih.gov/clinvar/).

Novel molecular variants were assessed by pathogenicity prediction tools: PolyPhen2 Mutation Assessor, SIFT, FATHMM, and MutationTaster softwares — for nucleotide changes localized in coding sequence and MaxEnt, NNSPLICE, or SSF — for nucleotide changes identified in intronic sequence, respectively. Additionally, the minor allele frequency (MAF) in different public databases (e.g., dbSNP, ESP6500, ExAC, gnomAD) was also included in this assessment.

Molecular data of four of our patients (two-generation family with MPSIVA) were mentioned earlier by other authors (Bunge et al. 1997; Tylki-Szymanska et al. 1998).

## Results

The mean age at diagnosis in the studied group was 6 years and ranges from 1 to 28 years. In 10/22, the first symptoms were noted around 12 months of life, and included: chest wall deformity or hip dislocation mostly. Skeletal deformation and growth retardation were the most constant features after infancy, noted around the age of three (Table 1).

**Table 1:**
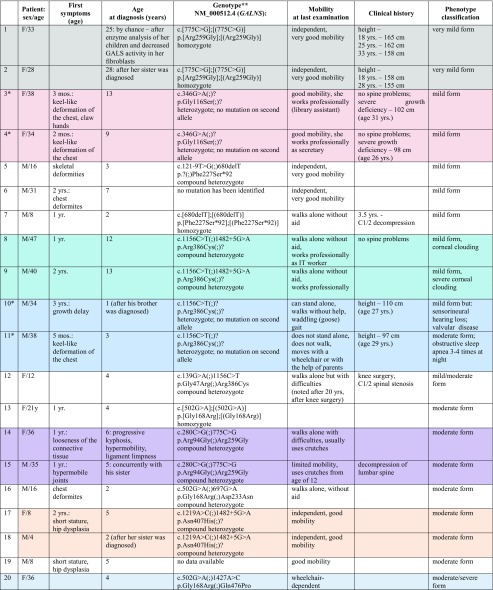

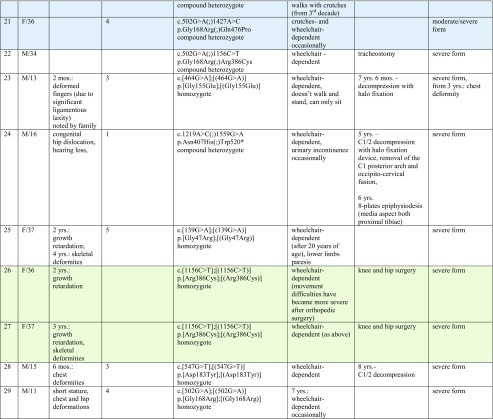
Clinical characteristics of 29 patients with MPS IVA (siblings are marked in colors; patients 14 and 15 are descendants of patient 1)

*Patients from Belarus

**The nomenclature of molecular variants follows the Human Genome Variation Society guidelines (HGVS, www.hgvs.org/mutnomen) according to *GALNS* reference sequence NM_000512.4 and NP_000503.1, followed the Human Gene Mutation Database (HGMD Professional, https://portal.biobase-international.com/hgmd/) and ClinVar (https://www.ncbi.nlm.nih.gov/clinvar/). Additionally, protein effect was predicted for identified variants, if it is possible

### Clinical outcome

Based on onset of motor impairment (0–20 years, > 20 and > 30 years) the patients were classified as mild, moderate, and severely affected (respectively). Moreover, we take also ocular and heart involvement or respiratory complications into consideration.

As presented in Table 1, mild (attenuated) phenotype was the most frequent (11 cases), another ten patients corresponded to severe or severe/moderate course of the disease, and eight were classified as moderate or mild/moderate. At the time of last examination, 13 affected classified as severe were unable to walk independently, and had to use the wheelchair (11/29 cases) and (or) crutches (4/29). Eight patients underwent chirurgical procedures for orthopedic reasons (4/8) and (or) spine anomalies (6/8). Respiratory decline was noted in two affected, of whom one needed tracheostomy, and another suffered from obstructive sleep apnea. Of the other symptoms, corneal clouding (2/29), valvular disease (1/29), and sensorineural hearing loss (1/29) were observed.

#### Height and weight assessment

The values of body length and weight were pooled from documentation of the periodic screening that is conducted for each child in Poland, and from patients’ medical files from CMHI. The mean values for body length and weight at birth of 24 patients are presented in Table 2. The mean values for boys were 3719.3 g and 57 cm, while for girls − 3292 g and 54.7 cm, respectively. The body length at birth was statistically greater for groups of girls and boys than in the general population (Fenton and Kim 2013). For birth body weight, the differences were not statistically significant.

**Table 2:** Body length and weight of MPSIVA patients at birth

		Body weight (g)	Body length (cm)	*p* value for weight*	*p* value for length*
Study group	Boys (*n* = 10)	3719 ± 461	57.3 ± 3.34	0.35	0.003
	Girls (*n* = 4)	3292 ± 550	54.7 ± 3.47	0.55	0.002
Health population	Boys	3500 ± 0.6	52.2 ± 2.8		
	Girls	3400 ± 0.5	51.3 ± 2.5		

**Fig. 1:**
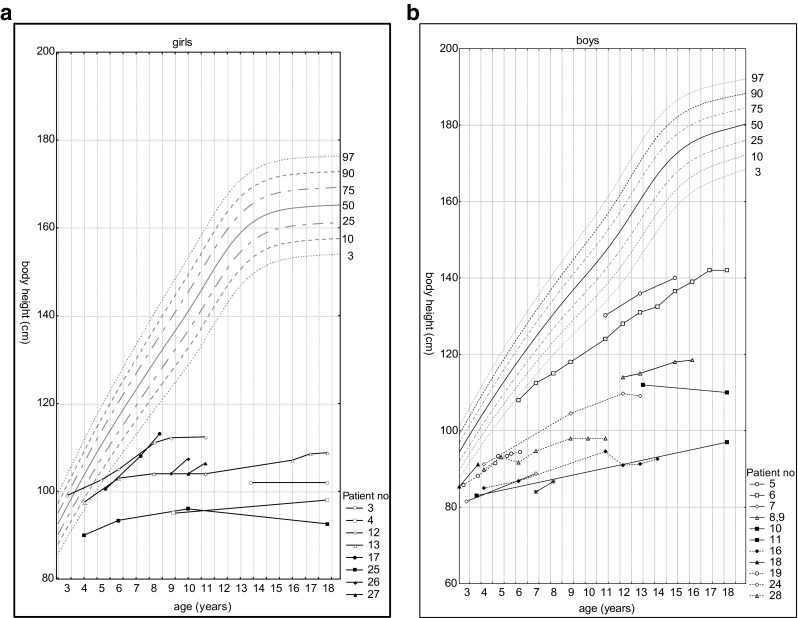
Height development in females (A) and males (B) with MPSIVA in comparison to percentile charts for healthy population

Data of height development in comparison to general population are shown per sex in Fig. 1 (A and B). Postnatal growth relatively normal during the first months of life thereafter began to slow down. In boys, the body height below the third percentile was reached after the 24th month of life, while in girls between the fourth and fifth years of life.

**Fig. 2:**
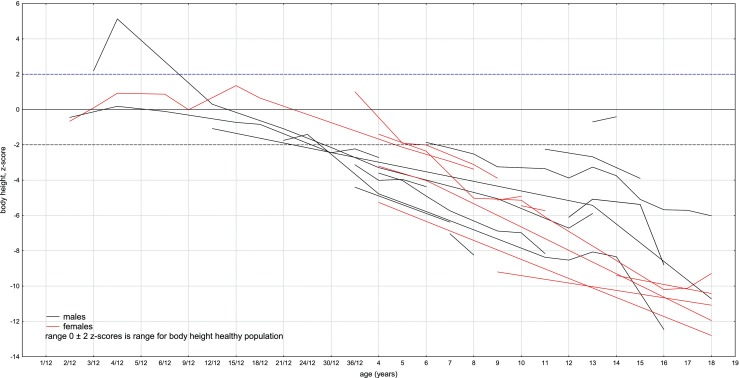
Height development in our MPS IVA group compared to the general population (females are in red, males are in black)

As noted in Fig. 2, the growth rate declined faster in boys than in girls—all male probands under 3 years of age were < 3 percentiles, while in age-matched females, exceptions were observed.

#### Head circumference assessment

**Fig. 3:**
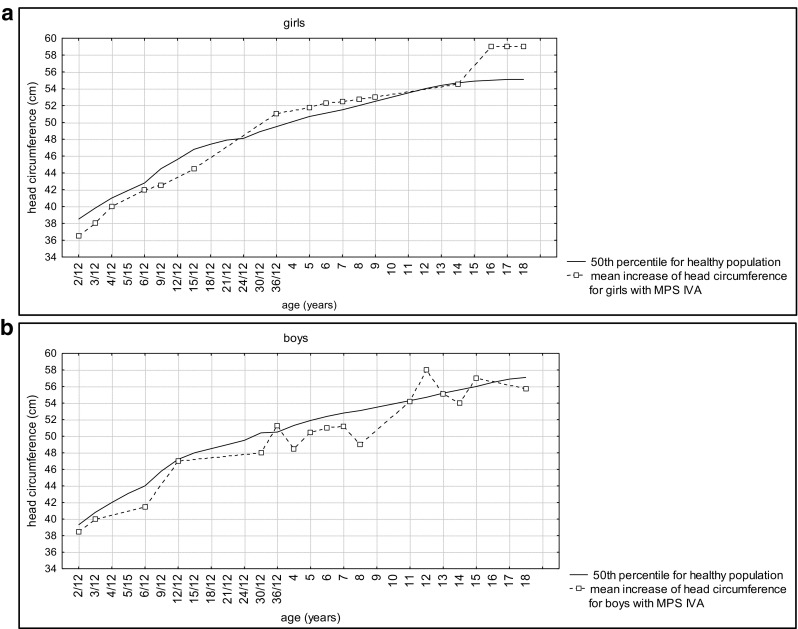
Head circumference (OFC) development in females (A) and males (B) with MPS IVA

In this study, head circumference for boys and girls was nearly 50th percentile to 15 years of age, when the mean head circumference for girls increased to above 75 percentile (Fig. 3A, B).

**Fig. 4:**
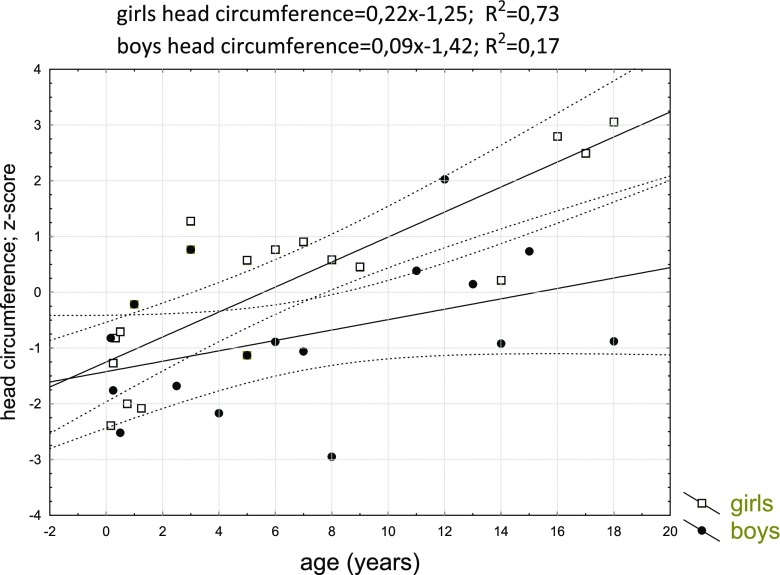
Linear regression of head circumferences. Table 3. Characteristics of the parameters of equations of straight line regression for patients with MPS IV, regression of the standardized head circumference against age

To show the directional changes in the growth of head circumference in patients with MPS IVA, the linear regression model was used. The statistically significant positive tendency of growth for the head circumference for girls (*r* = 0.85, *p* = 0.00) was found. For boys, correlation was *r* = 0.42, *p* = 0.10 and this observation was not statistically significant. To compare linear regression for boys and girls, comparison of correlation coefficient was made. The trend for growing of head circumference for girls was significantly higher than for boys. The difference between these two linear regressions was statistically significant (*p* = 0.04). Table 3 shows the characteristics of regression equations and Fig. 4 contains their graphic.

**Table 3:** Characteristics of the parameters of equations of straight line regression for patients with MPS IV, regression of the standardized head circumference against age

Head circumference	B_0_	R^2^	B_1_	p
Girls	0.22	0.73	− 1.25	*p* = 0,00
Boys	0.09	0.17	− 1.42	*p* = 0,00

#### Body and face proportion analyses

**Fig. 5:**
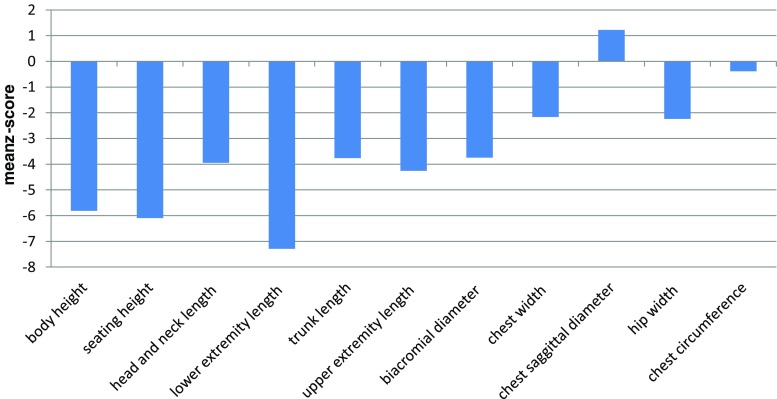
Mean z-scores for body proportions in MPSIVA patients

The results of body proportions are presented in Fig. 5. Patients’ statures were significantly marked by disproportion of trunk length (− 3.76 z-score) in relation to lower extremity length (− 7.26 z-score). At the same time, chest parameters were significantly different—chest circumference − 0.37 z-score and chest sagittal diameter 1.22 z-score.

**Fig. 6:**
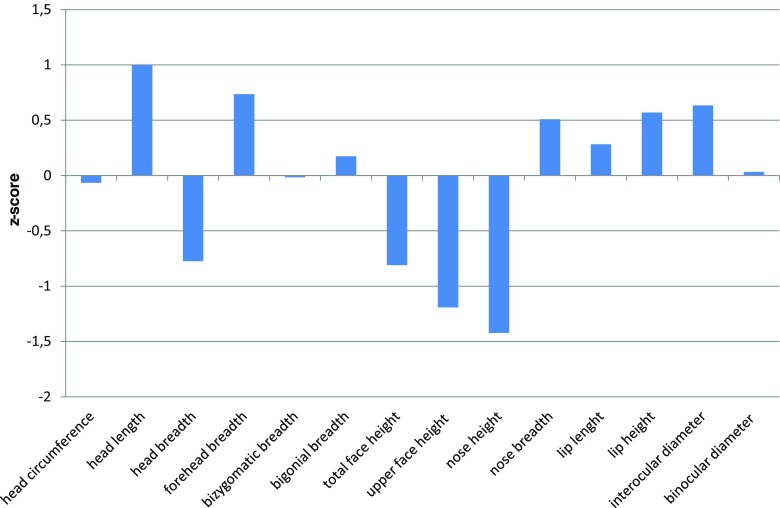
Facial anthropometry

As we presented in Fig. 6, some specificity in craniometry can be also noted. Namely, head was a little bit longer (0.99 z-score; 75th percentile) and narrowed (− 0.77 z-score; 10th percentile) than in general population, while face (− 1.21 z-score) and nose heights (− 1.41 z-score) were shorter than in healthy peers. The other facial parameters were normal.

### Pathogenic variants in *GALNS*

**Fig 7:**
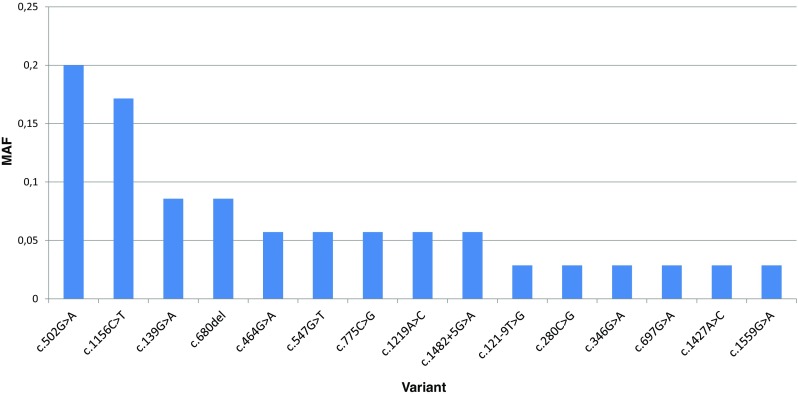
Results of mutation analysis in MPSIVA patients from Poland and Belarus

These molecular data were available for 27 patients (one did not have molecular diagnostics, in another no mutation in *GALNS* has been identified, and the parents have not decided for further testing). In four individuals from two families, affected mutations were identified only in one allele. Ten patients in the analyzed group were homozygous, 13 compound heterozygous for the *GALNS* mutation (Fig. 7).

In total, 15 different molecular variants on 35 alleles of *GALNS* gene among unrelated patients have been identified (Fig. 4). The c.502G>A was the most frequent (7/35, 20%), followed by c.1156C>T (6/35, 17%). The frequency of other variants was significantly lower (each < 10%). Five variants were novel, not reported to date in the literature and HGMD database. These were two missense substitutions: c.547G>T (p.Asp183Tyr) in patient 28 (homozygous) and c.1427A>C (p.Gln476Pro) in patients 20 and 21 (siblings), one single nucleotide deletion c.680del leading to frame shift and premature termination of the translation (p.Phe227Ser*92) in two families (individuals 5 and 7), two others were splice site changes, c.121-9T>G in acceptor site downstream in patient 5, and c.1482 + 5G>A in donor site upstream in two families—patients 8 and 9. All novel changes were predicted by in silico analysis to be pathogenic variants and were not annotated in the population database (MAF = 0).

## Discussion

### Clinical outcome

In majority of mucopolysaccharidosis type IVA patients, no distinctive clinical findings are noted at birth. Problems appear with age, but differ depending on the disease severity. The most severe form is usually apparent between 1 and 3 years. However, based on our clinical experience we may propose the thesis that if the disease would be suspected, the parents would notice any warring symptoms earlier. In contrary, the slow progressive (attenuated) one may not become evident until late childhood or even adolescence (Montaño et al. 2007). Our two-generation family may serve as an example for the latter. The diagnosis of MPSIVA in two adult cases (patients 1 and 2) was possible only after establishing a diagnosis in the probands (patients 14 and 15). One should, however, keep in mind that the classification is clinically based and hence affected individuals may change their status with age, especially as regards mobile ability. If the disability begins after surgery procedure (what we did observe in our probands) is it still in mild form or was it mild but graduate to moderate?

The severe form is characterized by disproportionate short stature, marked by pectus carinatum and short trunk, spine deformity, knock knee (genu valgum), resulting with waddling gait, and hypermobile wrist, finger joints, and ligamentous laxity (Montaño et al. 2007). Dens hypoplasia, accompanied by ligamentous laxity can lead to atlantoaxial instability and manifests as myelopathy. Moreover, it may subsequently lead to spinal canal stenosis and spinal cord compression (Solanki et al. 2013). Therefore, patients may be confined to wheelchairs even by their second decade of life. In some, especially in childhood age, cervical stabilization is used to prevent dislocations. Moreover, as a consequence of narrow, floppy airways (caused by keratan sulfate and chondroitin-6-sulfate accumulations along airways) and the restrictive effects of skeletal anomalies, respiratory problems often develop, and respiratory failure has been shown to be the primary cause of death in up to 63% of patients (Lavery and Hendriksz 2015). Today we cannot support the latter observation. Among our cases, respiratory decline was not a frequent feature. It may, however, result from both—mild form of the disease (which dominate in studied group), as well as young age of many of our patients.

Attenuated form of MPSIVA manifests, in contrast, with minor skeletal abnormalities or hip pain, and moderate short stature. Skeletal findings worsen however over time. Adult patients from our group, who were classified as mild form, are generally in good physical condition—work at a professional level. Nevertheless, two siblings had very severe short stature (3 and 4 and 10 and 11), in comparison to others with higher growth (patients 8 and 9) or, especially, very mild disease presentation (patients 1 and 2) who, as an only health problem, noted “height loss” with age (as presented in Table 1).

Since abnormalities in anthropometric parameters are one of the earliest and most characteristic features of MPSIVA, what we proved in “Results”, our effort was to re-evaluate growth data of our patients considering the causative mutation. In both above-mentioned families (3 and 4 and 10 and 11) pathogenic variant in *GALNS* has been identified just in one allele, that is why we can only assume that c.346G>A and c.1156C>T may influence severe growth deficiency. It is however in line with previous studies where authors concluded on p.G116S and p.R386C defining the severe MPS IVA phenotype (Sukegawa et al. 2000; Tomatsu et al. 2004).

Moreover, looking at our two-generation family, we may conclude that the more severe phenotype observed in patients 14 and 15 was rather a result of paternally inherited variant (c.280G>C). The probands’ mother, a homozygote for c.775C>G (patient 1), had a very attenuated MPSIVA form.

### Anthropometric phenotype

#### Height

Apart from two sisters with very mild disease presentation, short stature was a constant feature in our group. Of note, birth parameters were within normal range in all patients, and growth deficits were observed later, especially over the age of 3. These results confirm the existence of a downward trend in degree and direction of deviation in children with MPS IVA when compared to reference charts. When compared to the general population, girls have lost about 10 cm during the first 5 years of life while boys in this same period lost about 18 cm. In subsequent years, the values of body height were getting lower in comparison with the reference charts. This showed pathological character of physical development of children with MPS IVA. In our study, boys reached third percentile faster than girls. We were unable to find any explanation. It may be an issue for further observation. It may be possible that, e.g., males are more sensitive for GAGs accumulation, as a result of specific *GALNS* variants, environmental component, any other genetic polymorphism, or ecosensitivity.

#### Skeletal anomalies and craniofacial dysmorphism

According to the guideline for the management of Morquio A syndrome, the head circumference should be assessed at diagnosis and at each visit to document the change in individuals over time (Hendriksz et al. 2015). The head circumferences usually are normal for age but in comparison with short stature and narrowed arms give the impression of being huge. In girls from the analyzed group, the increase in the head circumference seems to be more dynamic.

To show the directional changes in the growth of head circumference in patients with MPS IVA, the linear regression model was used. This method showed a distinct positive tendency of growth for the head circumference in the period from the first month to 18 years of life. The dynamic of head circumference increase was greater in a group of girls than in boys. As a result, the mean adult head circumference was greater among girls.

In boys, we would rather conclude about a stable permanent OFC increase in accordance with the age. We are obviously aware that it should be interpreted very carefully because of limited data in this developmental period, and notwithstanding the measurements in girls are comparable to the boys in the subsequent stages of development. Craniometric analyses showed that head circumference did not differ from healthy peers (− 0.06 z-score).

The most common deformity for MPS IVA presents in the lower extremities of the knee and ankle valgus (Neufeld and Muenzer 2001). In this study, a significant reduction of body height was shown in all patients because of short trunk and crooked lower extremities. The chest presented pectus carinatum deformities. Apart recognizable skeletal features of MPSIVA mentioned above, the disease might also be characterized by specific facial abnormalities. These comprise prominent forehead, large mandible (prognathism), and broad mouth with widely spaced teeth. Mentioned disproportion might be characteristic for MPS IVA facial phenotype, being a result of GAGs accumulations with influence on skeletal and connective tissue.

#### Molecular data and phenotype–genotype correlation

The correlation between MPSIVA clinical phenotype and causative *GALNS* mutations has been established as regards GALNS activity (Montaño et al. 2007; Morrone et al. 2014). It means that mutations and variants, which result in the lowest enzyme activity, are more common in individuals with rapidly progressive, severe disease form. In contrary, affected individuals with more conservative genetic changes that retain partial enzymatic activity are more likely to present milder or slow progressive phenotype (Yamada et al. 1998; Tomatsu et al. 2005).

As mentioned in “Results”, substitutions c.502G>A and c. 1156C>T were the most frequent *GALNS* mutations in our group (Table 1). Both result in missense effect. Such data are different from other studies, including other populations. For example, Tomatsu et al. (2004) found c. 1156C>T to have the highest frequency among unrelated patients from Latin America—32.5%.

The first is a transition from G to A in exon 5 which causes substitution of highly conserved amino acid—Gly by Arg at 168 protein position (p.Gly168Arg) and damages sulfatase domain. This variant was reported previously as a mutation causing MPSIVA (HGMD Accession CM970588, ClinVar RCV000633457.1) by Bunge et al. (1997) and Nykamp et al. (2017). It was also annotated in ExAC database with MAF = 0.000016. The second is a transition from C to T in exon 11 which results in substitution of highly conserved amino acid, Arg by Cys at 386 protein position (p.Arg386Cys) and damages alkaline-phosphatase-like, core domain. This variant was also reported previously as a mutation causing MPSIVA (HGMD Accession CM950540, ClinVar RCV000000735.7) by Ogawa et al. (1995) and Tomatsu et al. (2004). It was annotated in gnomAD database with MAF = 0.000083.

Other variants have been identified only in single families from our cohort (c.280C>G, c.346G>A, c.697G>A, c.1559G>A), therefore it is difficult to assess their phenotypic correlation. Considering our affected patients in whom novel *GALNS* pathogenic variants have been identified, two were homozygous for c.547G>T (patient 28) and for c.680del (patient 7), thus we had a chance to establish possible genotype-phenotype correlation. Thus, patient 28 presented with severe MPSIVA course, with first symptoms noted at the age of 6 months, which was chest deformity, then cervical spine decompression and finally, wheelchair-dependence. It seems hence that the substitution c.547G>T (p.Asp183Tyr), within weakly conserved nucleotide, may cause severe and progressive form of MPSIVA. In contrary, the c.680delT (p.Phe227Ser*92) mutation, which in our patients resulted in mild disease presentation with the first symptoms noted around 1 year, and no fast progression to motoric disability (case 7). Given that the latter is deletion, which—in addition—include highly conserved amino acid, such a clinical observation is interesting. Namely, the deletion gives frameshift effect, hence the resulted phenotype is supposed rather to be severe.

#### Correlation between growth development and causative mutations in GALNS gene

Concerning growth phenotype and its possible correlation with genotype, we like to note that the mildest form of MPSIVA, manifesting, i.e., with normal height, was observed in sisters (patients 1 and 2), homozygous for c.280C>G (p.775C>G). Whereas among our pediatric patients, the significantly less severe height deficiency was observed in males 5 and 6. Their growth increase was progressive, and remained at the same level during time of observation (Fig. 1B). Unfortunately, in patient 6, sequencing of *GALNS* gene has not revealed any mutation (and the parents have not agreed for further testing to check for, i.e., indels), so the only proposed correlation as regards height was for c.121-9T>G(;)680delT mutations, resulting in our group in mild MPSIVA (Table 1). In contrary, the height was the lowest in patient 25 (Gly47Arg homozygous) (Fig. 1A). It slowly increased till the age of 10, when more significant decline was noted (continued to age 18 years). Finally, in the proband being homozygote with novel *GALNS* mutation (patient 28), growth declined around the age of 4 to 5, then increased from 6 to 9 years and maintained at the same level thereafter, during follow-up.

Moreover, worth to note is that in five of our male probands (6, 16, brothers 8, 9 and 24) about the age of 12 years, rapid increase in growth was noted (Fig. 5). That was not observed in any of our female cases. We are obviously aware that there are also other factors (despite genotype) that influence, discussed herein, growth development. It might be, e.g., result of growth spurt, but if so, why was it not noted among girls? Our hope is that further studies, including more patients with specific *GALNS* variant will provide novel evidence and verify our observations.

## Conclusion

Based on clinical re-evaluation of 29 patients with MPSIVA presented herein, with focus on anthropometric and molecular data, we like to underline the importance of detailed anthropometric assessment, which add important, objective clinical details, being valuable even for prediction of the phenotype. We do believe that such analyses, if performed as a rule in other rare inherited conditions as well, give novel insight into the phenotypic characteristics, and natural history of any of them. In our study, the following conclusions could be drawn:

1. body length at birth was statistically greater in children with this disorder than in the general population,
2. body height below the third percentile was reached earlier by boys than girls (after 24th month vs. fourth and fifth years of life); during the first 5 years of life, females lost about 10 cm while boys in this same period — about 18 cm,
3. dynamics of head circumference growth was grater in a group of girls than in boys, with mean head circumference in adulthood greater among girls,
4. although anthropometric deficiencies increased with age of MPSIVA patients, the height within normal range is however possible, depending on the causative *GALNS* variant.

Regarding the molecular data, apart from novel pathogenic variants identified in MPSIVA patients from Poland and Belarus (listed below), genotype-phenotype analyses showed that:

1. milder (attenuated) forms of mucopolysaccharidosis type IVA might result from substitution c.280C>G (p.775C>G) and c.680delT (p.Phe227Ser*92),
2. the less severe growth deficiencies were supposed to result from c.280C>G (p.775C>G) and may be observed in heterozygotes c.121-9T>G(;)680delT,
3. variant c.280C>G (p.Arg94Gly) influences more severe MPSIVA expression,
4. the most frequent variants in our central and eastern European population were c.502G>A (20%) and c.1156C>T (17%), moreover, five novel molecular variant in GALNS were identified — c.121-9T>G (p?), c.547G>T (p.Asp183Tyr), c.680del (p.Phe227Ser*92), c.1427A>C (p.Gln476Pro), and c.1482+5G>A (p?).

## References

[CR1] Baker E, Guo XH, Orsborn AM, Sutherland GR, Callen DF, Hopwood JJ, Morris CP (1993). The Morquio A syndrome (mucopolysaccharidosis IVA) gene maps to 16q24.3. Am J Hum Genet.

[CR2] Brailsford JF (1929). Chondro-osteodystrophy: roentgenographic and clinical features of child with dislocation of vertebrae. Am J Surg.

[CR3] Bunge S, Kleijer WJ, Tylki-Szymanska A, Steglich C, Beck M, Tomatsu S, Fukuda S, Poorthuis BJHM, Czartoryska B, Orii T, Gal A (1997). Identification of 31 novel mutations in the N-acetylgalactosamine-6-sulfatase gene reveals excessive allelic heterogeneity among patients with Morquio A syndrome. Hum Mutat.

[CR4] Fenton TR, Kim JH (2013). A systematic review and meta-analysis to revise the Fenton growth chart for preterm infants. BMC Pediatr.

[CR5] Hendriksz CJ, Berger KI, Giugliani R, Harmatz P, Kampmann C, Mackenzie WG, Raiman J, Villarreal MS, Savarirayan R (2015). International guidelines for the management and treatment of Morquio A syndrome. Am J Med Genet A.

[CR6] Lavery C, Hendriksz C (2015). Mortality in patients with morquio syndrome. JIMD Rep.

[CR7] Martin R, Saller K (1957). Lehrbuch der anthropologie.

[CR8] Montaño AM, Tomatsu S, Gottesman GS, Smith M, Orii T (2007). International Morquio A registry: clinical manifestation and natural course of Morquio A disease. J Inherit Metab Dis.

[CR9] Morquio L (1929). Sur une forme de dystrophie osseuse familiale. Arch Med Infants Paris.

[CR10] Morrone A, Caciotti A, Atwood R, Davidson K, Chaoyi D, Francis-Lyon P, Harmatz P, Mealiffe M (2014). Morquio A syndrome-associated mutations: a review of alterations in the GALNS gene and a new locus-specific database. Hum Mutat.

[CR11] Neufeld EU, Muenzer J, Scriver CR (2001). The mucopolysaccharidoses. The metabolic and molecular bases of inherited disease.

[CR12] Nykamp K, Anderson M, Powers M, Garcia J, Herrera B, Ho YY, Kobayashi Y, Patil N, Thusberg J, Westbrook M, Topper S, Invitae Clinical Genomics Group (2017). Sherloc: a comprehensive refinement of the ACMG-AMP variant classification criteria. Genet Med.

[CR13] Ogawa T, Tomatsu S, Fukuda S, Yamagishi A, Rezvi GM, Sukegawa K, Kondo N, Suzuki Y, Shimozawa N, Orü T (1995). Mucopolysaccharidosis IVA: screening and identification of mutations of the N-acetylgalactosamine-6-sulfate sulfatase gene. Hum Mol Genet.

[CR14] Palczewska L, Niedźwiecka Z (2001) Indicators of somatic development of children and youth inWarsaw. In: Instytut Matki i Dziecka (ed) Developmental Period Medicine. Warsaw, pp 1–120

[CR15] Poorthuis BJ, Wevers RA, Kleijer WJ, Groener JE, de Jong JG, van Weely S, Niezen-Koning KE, van Diggelen OP (1999). The frequency of lysosomal storage diseases in the Netherlands. Hum Genet.

[CR16] Solanki GA, Martin KW, Theroux MC, Lampe C, White KK, Shediac R, Lampe CG, Beck M, Mackenzie WG, Hendriksz CJ, Harmatz PR (2013). Spinal involvement in mucopolysaccharidosis IVA (Morquio-Brailsford or Morquio A syndrome): presentation, diagnosis and management. J Inherit Metab Dis.

[CR17] Tomatsu S, Dieter T, Schwartz IV, Sarmient P, Giugliani R, Barrera LA, Guelbert N, Kremer R, Repetto GM, Gutierrez MA, Nishioka T, Serrato OP, Montaño AM, Yamaguchi S, Noguchi A (2004). Identification of a common mutation in mucopolysaccharidosis IVA: correlation among genotype, phenotype, and keratan sulfate. J Hum Genet.

[CR18] Tomatsu S, Fujii T, Fukushi M, Oguma T, Shimada T, Maeda M, Kida K, Shibata Y, Futatsumori H, Montaño AM, Mason RW, Yamaguchi S, Suzuki Y, Orii T (2013). Newborn screening and diagnosis of mucopolysaccharidoses. Mol Genet Metab.

[CR19] Tomatsu S, Montaño AM, Nishioka T, Gutierrez MA, Peña OM, Tranda Firescu GG, Lopez P, Yamaguchi S, Noguchi A, Orii T (2005). Mutation and polymorphism spectrum of the GALNS gene in mucopolysaccharidosis IVA (Morquio A). Hum Mutat.

[CR20] Tomatsu S, Montaño AM, Oikawa H, Smith M, Barrera L, Chinen Y, Thacker MM, Mackenzie WG, Suzuki Y, Orii T (2011). Mucopolysaccharidosis type IVA (Morquio A disease): clinical review and current treatment. Curr Pharm Biotechnol.

[CR21] Tylki-Szymanska A, Czartoryska B, Bunge S, van Diggelen OP, Kleijer WJ, Poorthuis BJHM, Huijmans JGM, Gorska D (1998). Clinical, biochemical and molecular findings in a two-generation Morquio A family. Clin Genet.

[CR22] Sukegawa K, Nakamura H, Kato Z, Tomatsu S, Montaño AM, Fukao T, Toietta G, Tortora P, Orii T, Kondo N. Biochemical and structural analysis of missense mutations in N-acetylgalactosamine-6-sulfate sulfatase causing mucopolysaccharidosis IVA phenotypes (2000). Hum Mol Genet 22(9): 1283–1290 10.1093/hmg/9.9.1283 10814710

[CR23] Yamada N, Fukuda S, Tomatsu S, Muller V, Hopwood JJ, Nelson J, Kato Z, Yamagishi A, Sukegawa K, Kondo N, Orii T (1998). Molecular heterogeneity in mucopolysaccharidosis IVA in Australia and Northern Ireland: nine novel mutations including T312S, a common allele that confers a mild phenotype. Hum Mutat.

